# Update: Demographic, Product, and Substance-Use Characteristics of Hospitalized Patients in a Nationwide Outbreak of E-cigarette, or Vaping, Product Use–Associated Lung Injuries — United States, December 2019

**DOI:** 10.15585/mmwr.mm6849e1

**Published:** 2019-12-13

**Authors:** Matthew J. Lozier, Bailey Wallace, Kayla Anderson, Sascha Ellington, Christopher M. Jones, Dale Rose, Grant Baldwin, Brian A. King, Peter Briss, Christina A. Mikosz, Lung Injury Response Epidemiology/Surveillance Task Force, Lung Injury Response Epidemiology/Surveillance Task Force, Chelsea Austin, Sharyn Brown, Gyan Chandra, Angela Coulliette-Salmond, Kelsey Coy, Dustin Currie, Alissa Cyrus, Melissa Danielson, Geroncio Fajardo, Allison Gately, Sonal Goyal, Sierra Graves, Janet Hamilton, Donald Hayes, Denise Hughes, Mia Israel, Michael Landen, Ruth Lynfield, Suzanne Newton, Rashid Njai, Loria Pollack, Jeff Ratto, Matthew Ritchey, Katherine Roguski, Phillip Salvatore, Stephen Soroka, Elizabeth Swedo, Kimberly Thomas, Stephanie Thomas

**Affiliations:** ^1^National Center for Emerging and Zoonotic Infectious Diseases, CDC; ^2^National Center on Birth Defects and Developmental Disabilities, CDC; ^3^Oak Ridge Institute for Science and Education, Oak Ridge, Tennessee; ^4^National Center for Chronic Disease Prevention and Health Promotion, CDC; ^5^National Center for Injury Prevention and Control, CDC.; National Center for Environmental Health, CDC; National Center for Chronic Disease Prevention and Health Promotion, CDC; National Center for Chronic Disease Prevention and Health Promotion, CDC; National Center for Emerging and Zoonotic Infectious Diseases, CDC; National Center for Chronic Disease Prevention and Health Promotion, CDC; Center for Global Health, CDC; Office of Minority Health and Health Equity, CDC; National Center on Birth Defects and Developmental Disabilities, CDC; National Center for Emerging and Zoonotic Infectious Diseases, CDC; National Center for Injury Prevention and Control, CDC; National Center for Chronic Disease Prevention and Health Promotion, CDC; National Center on Birth Defects and Developmental Disabilities, CDC; , Council of State and Territorial Epidemiologists; National Center for Chronic Disease Prevention and Health Promotion, CDC; National Center for Immunization and Respiratory Diseases, CDC; Council of State and Territorial Epidemiologists; New Mexico Department of Health; Minnesota Department of Health; National Center on Birth Defects and Developmental Disabilities, CDC; Deputy Director for Non-Infectious Diseases, Office of the Director, CDC; National Center for Chronic Disease Prevention and Health Promotion, CDC; Center for Surveillance, Epidemiology, and Laboratory Services, CDC; National Center for Chronic Disease Prevention and Health Promotion, CDC; National Center for Emerging and Zoonotic Infectious Diseases, CDC; National Center for Injury Prevention and Control, CDC; National Center for Emerging and Zoonotic Infectious Diseases, CDC; National Center for Injury Prevention and Control, CDC; Center for Surveillance, Epidemiology, and Laboratory Services, CDC; National Center for Immunization and Respiratory Diseases, CDC.

CDC, the Food and Drug Administration (FDA), state and local health departments, and public health and clinical stakeholders continue to investigate a nationwide outbreak of e-cigarette, or vaping, product use–associated lung injury (EVALI) (*1*). This report updates demographic and self-reported product-use and substance-use characteristics of hospitalized EVALI patients reported to CDC from available interview or medical record abstraction data. As of December 3, 2019, all 50 states, the District of Columbia (DC), and two U.S. territories (Puerto Rico and U.S. Virgin Islands) reported 2,291 patients hospitalized with EVALI. A total of 48 (2% of total reported cases) deaths occurred in 25 states and DC. Median patient age was 24 years, 67% were male, and the largest number of weekly hospitalized cases occurred during the week of September 15, 2019; weekly hospitalized cases since then have steadily declined. Among all hospitalized EVALI patients reported to CDC weekly, the percentage of recent cases (patients hospitalized within the preceding 3 weeks) declined from 58% reported November 12 to 30% reported December 3. Overall, 80% of hospitalized EVALI patients reported using tetrahydrocannabinol (THC)-containing e-cigarette, or vaping, products. “Dank Vapes,” a class of largely counterfeit THC-containing products of unknown origin, were the most commonly reported THC-containing branded products nationwide and among all major U.S. Census regions. However, regional differences in THC-containing product use were noted; TKO and Smart Cart brands were more commonly reported by patients in the West region compared with other regions. Because most patients reported using THC-containing products before symptom onset, CDC recommends that persons should not use e-cigarette, or vaping, products that contain THC. The nationwide diversity of THC-containing products reported by patients suggests it is unlikely a single brand is responsible for the EVALI outbreak, and regional differences in THC-containing products might be related to product sources. Although it appears that vitamin E acetate is associated with EVALI, many substances and product sources are being investigated, and there might be more than one cause. Therefore, while the investigation continues, persons should consider refraining from the use of all e-cigarette, or vaping, products.

CDC has worked with state health departments and a task force formed by the Council of State and Territorial Epidemiologists to develop and disseminate surveillance case definitions* and data collection tools^†^ to monitor and track cases beginning in August 2019. States and jurisdictions voluntarily report the number of confirmed and probable hospitalized EVALI cases and all EVALI-associated deaths to CDC on a weekly basis. This report is limited to data on hospitalized EVALI patients and all EVALI-associated deaths reported to CDC as of December 3, 2019 (*2*), and updates patient demographic characteristics, the number and diversity of self-reported substances, and brands used in e-cigarette, or vaping, products. Distribution of THC-containing brands is reported nationally and by U.S. Census region.^§^ 2018 U.S. Census population estimates were used to calculate rates (hospitalized EVALI cases per 1 million population) by state.^¶^ Because of the time required to investigate cases, weekly reports to CDC include recent EVALI cases (patients hospitalized within the preceding 3 weeks) and past EVALI cases (those hospitalized earlier). To assess the recent trajectory of the EVALI outbreak, this report examined the percentage of all hospitalized EVALI patients reported weekly who had been hospitalized within the preceding 3 weeks.

As of December 3, 2019, all 50 states, DC, Puerto Rico, and the U.S. Virgin Islands reported 2,291 hospitalized EVALI cases to CDC (Table). Overall, a total of 48 (2% of total reported cases) EVALI-associated deaths occurred in 25 states and DC, which include one nonhospitalized case and two cases with unknown hospitalization status. Among hospitalized EVALI patients for whom data were available, 67% were male, and the median age was 24 years (range = 13–77 years); 78% of patients were aged <35 years and 16% were <18 years. Most EVALI patients were non-Hispanic white (75%), and 16% were Hispanic. Among the 48 deaths, 54% of patients were male, and the median age was 52 years (range = 17–75 years).

**TABLE:** Demographic and e-cigarette, or vaping, product use characteristics among patients with hospitalized* cases of e-cigarette, or vaping, product use–associated lung injury (EVALI) reported to CDC — United States, August–December 2019^†^

Characteristic	All EVALI patients, No./Total no. (%)^§^ (N = 2,291)	Any THC-containing product use (n = 1,421)	Any nicotine-containing product use (n = 956)	Any CBD-containing product use (n = 214)
**Sex**				
Male	1,449/2,155 (67)	987/1,414 (70)	645/952 (68)	135/213 (63)
Female	706/2,155 (33)	427/1,414 (30)	307/952 (32)	78/213 (37)
**Median age, yrs (range)**	24 (13–77)	23 (13–77)	22 (13–75)	27 (14–70)
**Age group (yrs)**				
13–17	341/2,159 (16)	237/1,417 (17)	177/953 (19)	16/213 (8)
18–24	817/2,159 (38)	567/1,417 (40)	424/953 (45)	72/213 (34)
25–34	524/2,159 (24)	341/1,417 (24)	199/953 (21)	64/213 (30)
35–44	278/2,159 (13)	171/1,417 (12)	95/953 (10)	36/213 (17)
45–64	165/2,159 (8)	88/1,417 (6)	46/953 (5)	24/213 (11)
≥65	34/2,159 (2)	13/1,417 (1)	12/953 (1)	1/213 (0)
**Race/Ethnicity^¶^**				
White	1,135/1,521 (75)	854/1,139 (75)	630/806 (78)	108/176 (61)
Black or African American	56/1,521 (4)	36/1,139 (3)	30/806 (4)	8/176 (5)
American Indian or Alaska Native	9/1,521 (1)	7/1,139 (1)	8/806(1)	4/176 (2)
Asian, Native Hawaiian, or other Pacific Islander	34/1,521 (2)	18/1,139 (2)	18/806 (2)	5/176 (3)
Other	39/1,521 (3)	32/1,139 (3)	24/806 (3)	6/176 (3)
Hispanic	248/1,521 (16)	192/1,139 (17)	96/806 (12)	45/176 (26)
**Case status**				
Confirmed	1,221/2,288 (53)	802/1,419 (57)	505/956 (53)	125/213 (59)
Probable	1,067/2,288 (47)	617/1,419 (43)	451/956 (47)	88/213 (41)
Substances used in e-cigarette or vaping products**^,††^				
Any THC-containing product use	1,421/1,782 (80)	1,421/1,421 (100)	713/956 (75)	172/214 (80)
Daily THC-containing product use	581/770 (75)	581/770 (75)	297/415 (72)	102/130 (78)
Any nicotine-containing product use	956/1,782 (54)	713/1,421 (50)	956/956 (100)	97/214 (45)
Daily nicotine-containing product use	482/568 (85)	351/416 (84)	482/568 (85)	68/79 (86)
Any CBD-containing product use	214/1,782 (12)	172/1,421 (12)	97/956 (10)	214/214 (100)
**Combination of substance use**				
Both THC- and nicotine-containing product use	713/1,782 (40)	713/1,421 (50)	713/956 (74)	81/214 (3)
Both THC- and CBD-containing product use	172/1,782 (10)	172/1,421 (12)	81/956 (8)	172/214 (80)
Both nicotine- and CBD-containing product use	97/1,782 (5)	81/1,421 (6)	97/956 (10)	97/214 (45)
All three (CBD, nicotine, and THC)	81/1,782 (5)	81/1,421 (6)	81/956 (8)	81/214 (38)
**Exclusive substance use**				
THC-containing product use only	617/1,782 (35)	617/1,421 (43)	—	—
Nicotine-containing product use only	227/1,782 (13)	—	227/956 (24)	—
CBD-containing product use only	26/1,782 (1)	—	—	26/214 (12)
No THC- or Nicotine- or CBD-containing product use	92/1,782 (5)	—	—	—

**Abbreviations:** CBD = cannabidiol; THC = tetrahydrocannabinol.

* Includes all hospitalized EVALI patients and EVALI-associated deaths regardless of hospitalization status.

^†^ For cases reported as of December 3, 2019.

^§^ Percentages might not sum to 100% because of rounding.

^¶^ Whites, blacks or African Americans, American Indians or Alaska Natives, Asians, Native Hawaiians or other Pacific Islanders, and Others were all non-Hispanic. Hispanic persons could be of any race.

** Data on both THC-containing and nicotine-containing product use required to be included (n = 1,782).

^††^ In the 3 months preceding symptom onset.

Since February 2019, the largest number of hospitalized EVALI patients (217) was reported during the week of September 15, 2019 (Figure 1). Since September 15, there has been a steady decline in hospitalized EVALI patients reported weekly to CDC. Among all hospitalized EVALI patients reported weekly to CDC by states since November 5, 2019, the percentage of recent EVALI cases declined from 58% reported November 12 to 30% reported December 3. Although EVALI cases have been reported in all states, DC, and two US territories, population-based prevalence rates varied widely across states (Figure 2).

**FIGURE 1:**
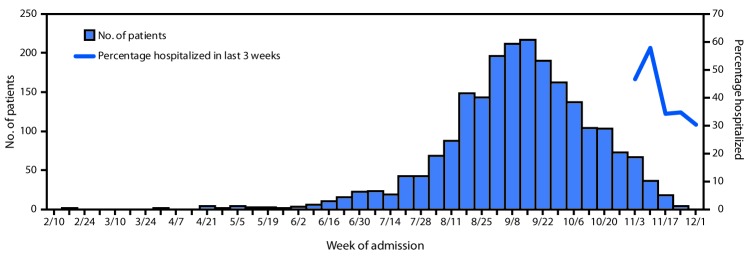
Number of patients (N = 2,163) with lung injury associated with e-cigarette, or vaping, product use, by week of hospital admission and percentage of patients hospitalized in last 3 weeks* — United States, February 10–December 3, 2019 * Percentage hospitalized within 3 weeks preceding the date reported to CDC.

**FIGURE 2:**
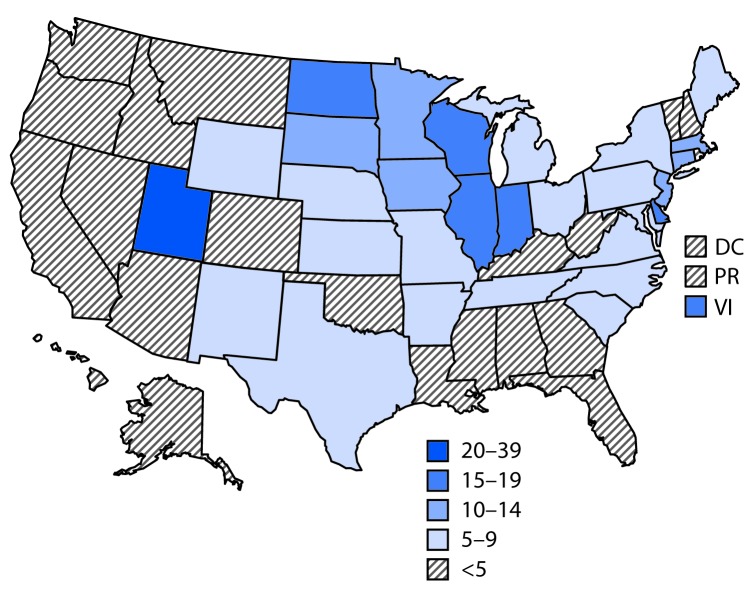
Prevalence* of hospitalized cases of e-cigarette, or vaping, product use–associated lung injury (N = 2,291) — United States, August–December 2019 The figure is a U.S. map, showing the prevalence (cases per 1 million) of hospitalized or deceased cases of e-cigarette, or vaping, product use–associated lung injury during August–December 2019. **Abbreviations:** DC = District of Columbia; PR = Puerto Rico; VI = U.S. Virgin Islands. * Number of cases per 1 million population. The U.S. Census population from 2010 was used to calculate prevalence for U.S. Virgin Islands, and U.S. Census population estimates from 2018 were used to calculate prevalence for all other states, the District of Columbia, and territories.

As of December 3, among 1,782 hospitalized EVALI patients with information on substances used in e-cigarette, or vaping, products in the 3 months preceding symptom onset, 80% and 35% reported any and exclusive use, respectively, of THC-containing products (Table). This compared with 54% and 13% of hospitalized EVALI patients who reported any and exclusive use, respectively, of nicotine-containing products and 12% and 1% who reported any and exclusive use, respectively, of cannabidiol (CBD)-containing products. Among 214 hospitalized patients who reported using CBD-containing products, 80% also reported using THC-containing products. Among 770 hospitalized patients who reported using THC-containing products and had frequency reported, 75% reported using THC-containing products daily.

Among hospitalized EVALI patients who reported using THC-containing e-cigarette, or vaping, products and had complete data on product use, 482 reported using 152 different products (861 observations; median = 2; range = 1–25). Dank Vapes, the most frequently reported product brand, was used by 56% of hospitalized EVALI patients nationwide (Figure 3). TKO (15%), Smart Cart (13%), and Rove (12%) were the next most commonly reported product brands. When stratified by U.S. Census regions, Dank Vapes remained the most commonly reported THC-containing product in all regions and was reported by >60% of hospitalized EVALI patients in the Northeast and South regions. Regional differences were seen in reported use of many products, including Smart Cart, which was reportedly used by a higher proportion of hospitalized EVALI patients in the West (24%) compared with those in the South (14%), Midwest (14%) and Northeast (6%). TKO was reported by more than twice as many hospitalized EVALI patients in the West (29%) as in the Northeast (14%), Midwest (12%), and South (2%) regions.

**FIGURE 3:**
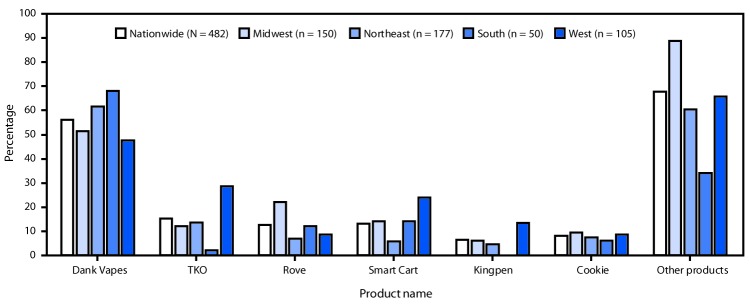
Percentage of hospitalized EVALI patients (N = 482) who reported brand names of THC-containing e-cigarettes, or vaping, products,* by U.S. Census region^†^ — United States, August–December 2019 **Abbreviations:** EVALI = e-cigarette, or vaping, product use–associated lung injury; THC = tetrahydrocannabinol. * ^“^Other products” included 146 unique products. Dabwood and Brass Knuckles were reported by 10% of patients in the Northeast and West regions. Off White, Moon Rocks, Chronic Carts, Mario Carts, Cereal Carts, Runtz, Dr. Zodiac, Eureka, Supreme G, and CaliPlug were reported by 1%–5% of patients nationwide. Use of 134 other products were reported by <1% of hospitalized patients. ^†^ *Northeast:* Connecticut, Maine, Massachusetts, New Hampshire, New Jersey, New York, Pennsylvania, Rhode Island, and Vermont. *Midwest:* Illinois, Indiana, Iowa, Kansas, Michigan, Minnesota, Missouri, Nebraska, North Dakota, Ohio, South Dakota, and Wisconsin. *South:* Alabama, Arkansas, Delaware, District of Columbia, Florida, Georgia, Kentucky, Louisiana, Maryland, Mississippi, North Carolina, Oklahoma, South Carolina, Tennessee, Texas, Virginia, and West Virginia. *West:* Alaska, Arizona, California, Colorado, Hawaii, Idaho, Montana, Nevada, New Mexico, Oregon, Utah, Washington, and Wyoming. Puerto Rico and U.S. Virgin Islands were included in the South region.

## Discussion

This report updates the characteristics of hospitalized EVALI patients, as well as those who died, and provides the first national data on the number and diversity of THC-containing products used. Among hospitalized EVALI patients as of December 3, 2019, the age, sex, and race distributions were similar to those reported previously (*1*–*3*), with a predominance of patients being young adults, male, and white. The persistent decline in number of cases reported each week since mid-September, coupled with the declining percentage of recent cases reported, suggest that the outbreak may have peaked around September 15. However, states continue to report new cases, including deaths, to CDC on a weekly basis. Therefore, this investigation remains ongoing, and it is important for states to remain vigilant with EVALI case finding and reporting.

THC-containing products continue to be the most commonly reported e-cigarette, or vaping, products used by hospitalized EVALI patients; 80% reported any use of these products in the 3 months preceding symptom onset. Dank Vapes were the most commonly reported THC-containing branded product reported nationally, as well as by U.S. Census region, which is consistent with data reported in October from Illinois and Wisconsin (*4*). Dank Vapes are a class of largely counterfeit THC-containing products and have been associated with EVALI (*4*,*5*). However, regional differences in THC-containing product use were identified. The nationwide diversity of THC-containing products reported by EVALI patients highlights that it is not likely a single brand that is responsible for the EVALI outbreak, and that regional differences in THC-containing products might be related to product sources.

The finding that most EVALI patients reported use of THC-containing products, in particular use of counterfeit branded products such as Dank Vapes, is important given recent findings from Minnesota that showed THC-containing products obtained from EVALI patients and counterfeit products seized in the state contained vitamin E acetate (*6*). Prior testing of bronchoalveolar lavage fluid samples implicated Vitamin E acetate as a chemical of concern in the outbreak after it was found in all assessed specimens from 29 EVALI patients (*7*). Additionally, FDA product testing identified vitamin E acetate in THC-containing products obtained from EVALI patients; among 545 THC-containing products collected from 70 EVALI patients, 79% of the 70 EVALI patients provided at least one THC-containing product, and among those, 76% provided at least one product containing vitamin E acetate.** However, given that a small but consistent number of EVALI patients report exclusive use of nicotine-containing (13%) or CBD-containing (1%) products (*1*,*2*), additional product and biologic testing from EVALI patients with these use patterns is warranted. Further research is being conducted by CDC and others to compare biologic specimens from EVALI patients with those from nonpatients who use e-cigarette, or vaping, products and to explore possible pathophysiologic mechanisms through which vitamin E acetate might cause lung injury.

The findings in this report are subject to at least five limitations. First, data on substances used in e-cigarette, or vaping, products were self-reported or reported by proxies (e.g., family members) and might be subject to recall or social desirability bias. Second, data related to product use were missing for many patients, and conclusions derived from these data might not be generalizable to the entire affected population. Third, many EVALI patients were not interviewed because of loss to follow-up, refusal to be interviewed, or lack of resources to conduct interviews, which might limit the generalizability of these findings to other EVALI patients. Fourth, reporting lags make it difficult to evaluate the trajectory of the outbreak during recent weeks. Finally, these data might be subject to misclassification of substance use for multiple reasons. Patients might not know the content of the e-cigarette, or vaping, products they used, and methods used to collect data regarding substance use varied across jurisdictions. CDC is working with state and federal partners (e.g., FDA) to link epidemiologic, product, and biologic samples to further explore the complexities of the EVALI outbreak.

Based on findings to date, CDC recommends that persons not use e-cigarette, or vaping, products that contain THC, especially those acquired from informal sources like friends, family members, or in-person or online dealers. In addition, persons should not add any other substances to products not intended by the manufacturer, including products purchased through retail establishments. Vitamin E acetate should not be added to e-cigarette, or vaping, products. However, although it appears that vitamin E acetate is associated with EVALI, many substances and product sources are being investigated, and there might be more than one cause. Therefore, while the investigation continues, persons should consider refraining from the use of all e-cigarette, or vaping, products. Adults using e-cigarette, or vaping, products to quit smoking should not return to smoking cigarettes; they should weigh all risks and benefits and consider using FDA-approved cessation medications.^††^ Adults who continue to use e-cigarette, or vaping, products should carefully monitor themselves for symptoms and see a health care provider immediately if they develop symptoms similar to those reported in this outbreak (*8*). Irrespective of the ongoing investigation, e-cigarette, or vaping, products should never be used by youths, young adults, or pregnant women.

**Summary**

**What is already known about this topic?**

Patients with e-cigarette, or vaping, product use–associated lung injury (EVALI) in Illinois and Wisconsin reported using a variety of tetrahydrocannabinol (THC)-containing products in the 3 months preceding illness; a product labeled “Dank Vapes” was most commonly reported.

**What is added by this report?**

Nationally, Dank Vapes were the most commonly reported THC-containing product by hospitalized EVALI patients, but a wide variety of products were reported, with regional differences. Data suggest the outbreak might have peaked in mid-September.

**What are the implications for public health practice?**

These data further support the association of EVALI with THC-containing products; it is unlikely that one brand is responsible for the outbreak. CDC recommends that persons not use e-cigarette, or vaping, products that contain THC.

## Footnotes

* https://www.cdc.gov/tobacco/basic_information/e-cigarettes/assets/2019-Lung-Injury-Surveillance-Case-Definition-508.pdf.

† https://www.cdc.gov/tobacco/basic_information/e-cigarettes/severe-lung-disease/healthcare-providers/pdfs/National-Case-Report-Form-v01.pdf.

§ *Northeast:* Connecticut, Maine, Massachusetts, New Hampshire, New Jersey, New York, Pennsylvania, Rhode Island, and Vermont. *Midwest:* Illinois, Indiana, Iowa, Kansas, Michigan, Minnesota, Missouri, Nebraska, North Dakota, Ohio, South Dakota, and Wisconsin. *South:* Alabama, Arkansas, Delaware, District of Columbia, Florida, Georgia, Kentucky, Louisiana, Maryland, Mississippi, North Carolina, Oklahoma, South Carolina, Tennessee, Texas, Virginia, and West Virginia. *West:* Alaska, Arizona, California, Colorado, Hawaii, Idaho, Montana, Nevada, New Mexico, Oregon, Utah, Washington, and Wyoming. Puerto Rico and U.S. Virgin Islands were included in the South region.

¶ https://www.census.gov/.

** https://www.fda.gov/news-events/public-health-focus/lung-illnesses-associated-use-vaping-products.

†† https://www.cdc.gov/tobacco/campaign/tips/quit-smoking/index.html?s_cid.
