# *Erratum:* Vol. 68, No. 49

**DOI:** 10.15585/mmwr.mm6904a7

**Published:** 2020-01-31

In the report “Update: Demographic, Product, and Substance-Use Characteristics of Hospitalized Patients in a Nationwide Outbreak of E-cigarette, or Vaping, Product Use–Associated Lung Injuries — United States, December 2019” on page 1444, there was an error in the Table.

In “TABLE. Demographic and e-cigarette, or vaping, product use characteristics among patients with hospitalized cases of e-cigarette, or vaping, product use–associated lung injury (EVALI) reported to CDC — United States, August–December 2019,” in the last column, under “Any CBD-containing product use,” in the first row below the row header “Combination of substance use,” the values for “Both THC- and nicotine-containing product use” should have read **81/214 (38**).

